# Supplementary figures and images for: Dr AFC: drug repositioning through anti-fibrosis characteristic
Source: Brief Bioinform. 2020 Jun 22;22(3):bbaa115. doi: 10.1093/bib/bbaa115 (PMC8138822; doi:10.1093/bib/bbaa115)

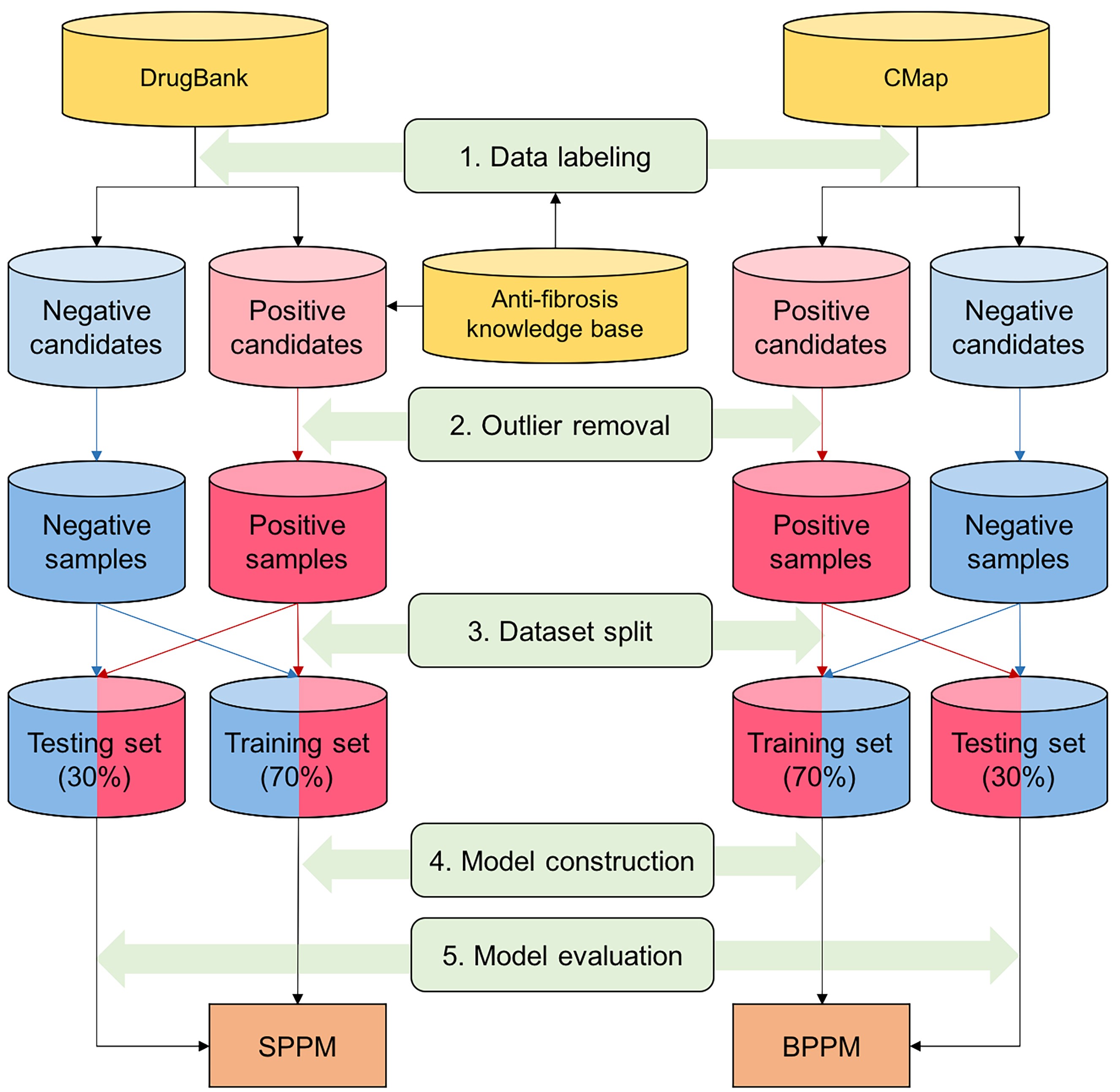

Supplement: Figure_S1_bbaa115 [file figure_s1_bbaa115.jpeg]

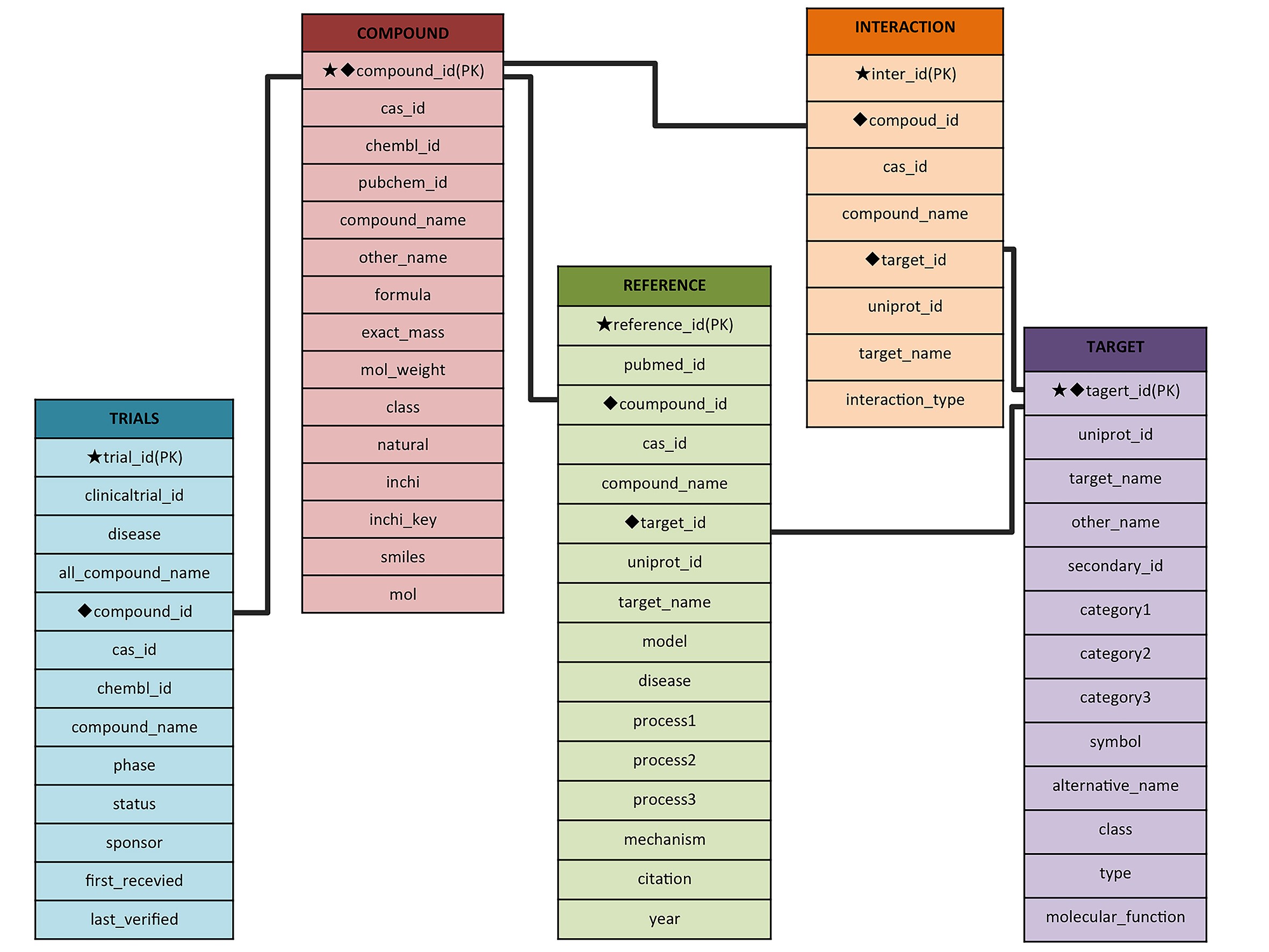

Supplement: Figure_S2_bbaa115 [file figure_s2_bbaa115.jpeg]

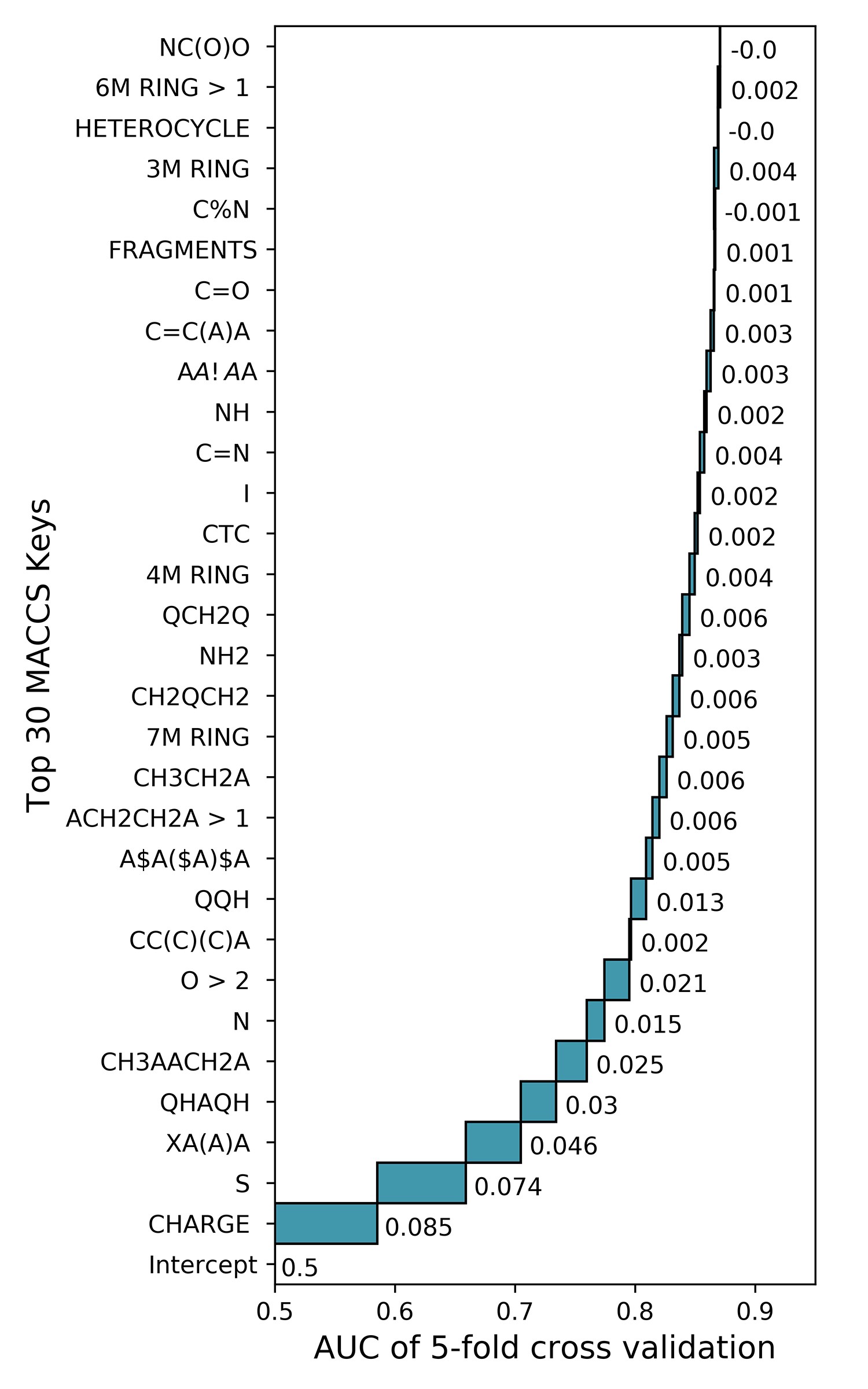

Supplement: Figure_S3_bbaa115 [file figure_s3_bbaa115.jpeg]
